# Supplementary material for: Novel Driver Strength Index highlights important cancer genes in TCGA PanCanAtlas patients
Source: PeerJ. 2022 Aug 11;10:e13860. doi: 10.7717/peerj.13860 (PMC9375969; doi:10.7717/peerj.13860)
Supplement: Supplemental Information 2 [file peerj-10-13860-s002.zip › COHORTS/genes plots/detailed/2021_8_16_14_9_distribution_events_detailed_females_41.pdf]

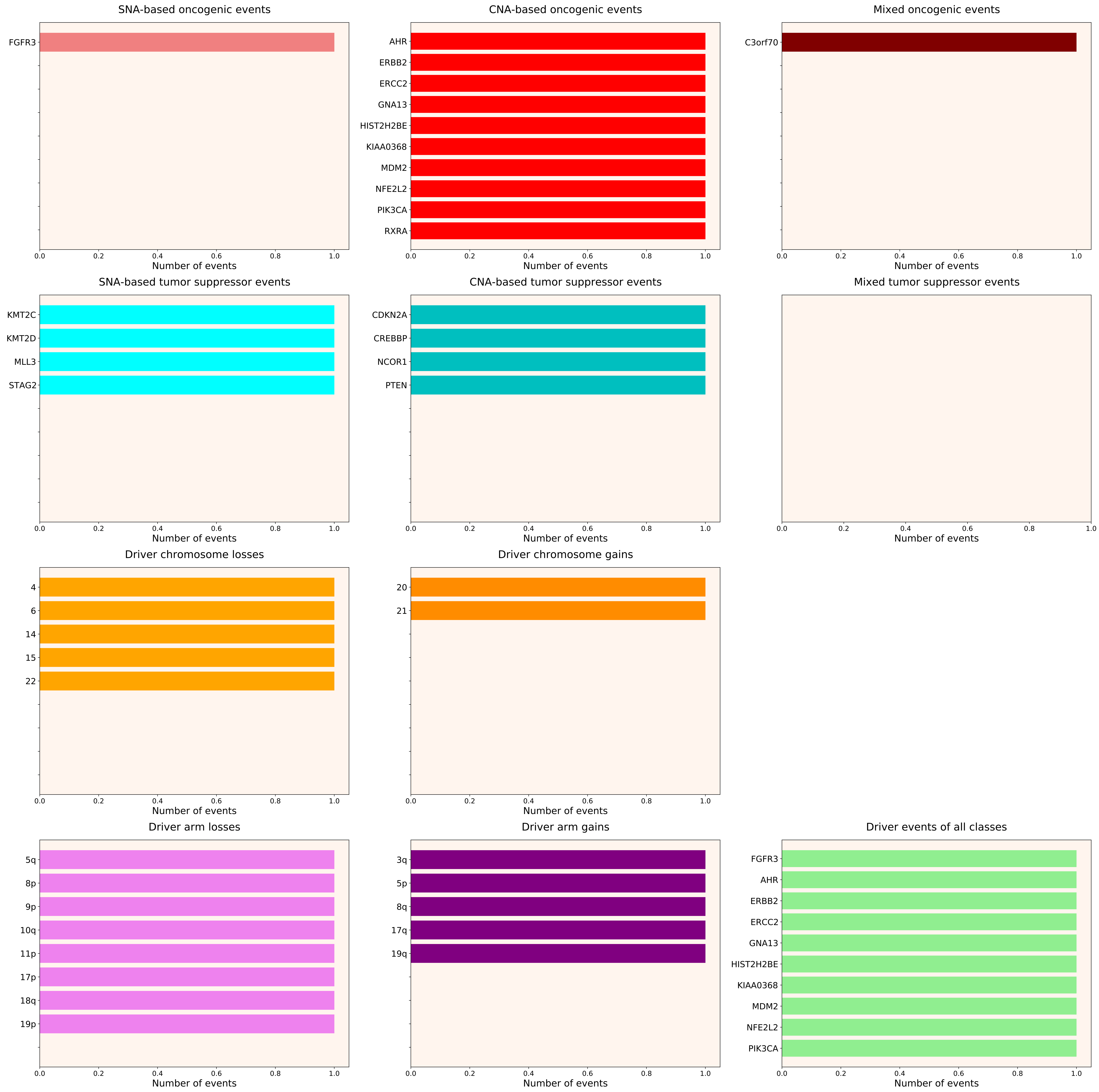

SNA-based oncogenic events
FGFR3
Number of events
CNA-based oncogenic events
AHR
ERBB2
ERCC2
GNA13
HIST2H2BE
KIAA0368
MDM2
NFE2L2
PIK3CA
RXRA
Number of events
Mixed oncogenic events
C3orf70
Number of events
SNA-based tumor suppressor events
KMT2C
KMT2D
MLL3
STAG2
Number of events
CNA-based tumor suppressor events
CDKN2A
CREBBP
NCOR1
PTEN
Number of events
Mixed tumor suppressor events
Number of events
Driver chromosome losses
4
6
14
15
22
Number of events
Driver chromosome gains
20
21
Number of events
Driver arm losses
5q
8p
9p
10q
11p
17p
18q
19p
Number of events
Driver arm gains
3q
5p
8q
17q
19q
Number of events
Driver events of all classes
FGFR3
AHR
ERBB2
ERCC2
GNA13
HIST2H2BE
KIAA0368
MDM2
NFE2L2
PIK3CA
Number of events
